# Mississippi Valley Medical Association

**Published:** 1888-09

**Authors:** 


MISSISSIPPI VALLEY MEDICAL ASSOCIATION.—Dr. J. Lucius Gray, the able and efficient Secretary of the Mississippi Valley Medical Association informs us that it meets at St. Louis, September 11. 12 and 13. The progrmme includes many papers and discussions of importance. The first day will be given to the discussion of "Abdominal Surgery." The second day to "Infant Feeding" and some obstetric subject. The third will be taken up with volunteer papers and some neurological subject. The Society cordially invites all members of the profession to be present.

